# Multi-Omics Analysis Reveals the Toxicity of Polyvinyl Chloride Microplastics toward BEAS-2B Cells

**DOI:** 10.3390/toxics12060399

**Published:** 2024-05-30

**Authors:** Chengzhi Liu, Shuang Chen, Jiangliang Chu, Yifan Yang, Beilei Yuan, Huazhong Zhang

**Affiliations:** 1 College of Safety Science and Engineering, Nanjing Tech University, Nanjing 210009, China; 18005194475@163.com (C.L.); chenshuangs28@163.com (S.C.); 18734001211@163.com (J.C.); yangyifan917@163.com (Y.Y.); 2 Department of Emergency Medicine, The First Affiliated Hospital of Nanjing Medical University, Nanjing 210029, China; 3 Institute of Poisoning, Nanjing Medical University, Nanjing 211100, China

**Keywords:** microplastics, polyvinyl chloride, BEAS-2B, multi-omics, lipid metabolism

## Abstract

Polyvinyl chloride microplastics (PVC-MPs) are microplastic pollutants widely present in the environment, but their potential risks to human lung health and underlying toxicity mechanisms remain unknown. In this study, we systematically analyzed the effects of PVC-MPs on the transcriptome and metabolome of BEAS-2B cells using high-throughput RNA sequencing and untargeted metabolomics technologies. The results showed that exposure to PVC-MPs significantly reduced the viability of BEAS-2B cells, leading to the differential expression of 530 genes and 3768 metabolites. Further bioinformatics analyses showed that PVC-MP exposure influenced the expression of genes associated with fluid shear stress, the MAPK and TGF-β signaling pathways, and the levels of metabolites associated with amino acid metabolism. In particular, integrated pathway analysis showed that lipid metabolic pathways (including glycerophospholipid metabolism, glycerolipid metabolism, and sphingolipid metabolism) were significantly perturbed in BEAS-2B cells following PVC-MPs exposure. This study provides new insights and targets for a deeper understanding of the toxicity mechanism of PVC-MPs and for the prevention and treatment of PVC-MP-associated lung diseases.

## 1. Introduction

Plastic products are widely used in a variety of fields, including healthcare, construction, and textiles, due to their low weight, durability, ease of processing, and low cost [1,2,3,4]. The global production of plastics is increasing dramatically each year, reaching 400 million tons in 2020, a figure that is expected to double over the next 20 years [5,6]. Because plastics typically take hundreds to thousands of years to degrade, they tend to accumulate in the environment, stemming from various sources [7,8]. Microplastics (MPs) are plastic particles with a diameter of less than 5 mm [9] that may come from the natural decomposition of plastic waste [10] or the use of daily necessities [11]. Reports from the World Health Organization (WHO) indicate the ubiquitous presence of microplastics in the ocean, air, soil, food, and beverages [12]. This could have long-term impacts on the environment and human health, creating a global cause for concern [13].

Currently, most studies have focused on microplastics in the marine environment, while relatively few studies have been conducted on atmospheric MPs [14]. Recently, attention has been focused on atmospheric microplastics, especially in light of concerns about human lung health and exposure outcomes. It has been reported that the per capita inhalation of 26–130 MPs particles per day from the air can pose a significant health risk to humans, especially for vulnerable groups like newborns and children [15]. Some exposure models have shown that moderately active males inhale up to 272 MPs particles per day [16]. After entering the human body, MPs may cause a number of chronic respiratory diseases [17]; affect gastrointestinal peristalsis [18]; and deposit on the surface of tissues or within cells, stimulating an inflammatory response, which can threaten human health [19]. Polyvinyl chloride (PVC), a prominent type of MP, is extensively used in toys, food packaging and cling film, squeeze bottles, shampoo bottles, detergent and cleaner bottles, medical supplies, construction products, etc. [20], with rising atmospheric levels due to atmospheric transport [21]. Recent studies have shown that exposure to PVC affects liver function, intestinal flora, lipid metabolism, and oxidative stress [22,23]. However, the molecular mechanisms underlying PVC-MP-induced cytotoxicity remain largely unknown.

Finite-element computer simulation approaches [24] and nanotechnology techniques [25] have been used to monitor the distribution and behavior of microplastics in the environment. However, these techniques have certain limitations, such as the need for large datasets associated with computationally costly resources or the complexity of the calibration step prior to data collection, respectively. In the face of these challenges, high-throughput techniques offer new solutions. Compared to traditional methods, high-throughput techniques are able to process large numbers of samples much more quickly, thus enabling the systematic analysis of toxicants in toxicology. Transcriptomics can identify alterations in total transcripts and screen key genes and pathways under stress [26]. Metabolomics allows the study of small-molecule metabolites and chemical reactions in cells or organisms, reflecting cellular physiology and revealing the biochemical dimension of biological information [27]. Metabolomics is the most accurate phenotypic-histologic approach and contains all the information on genetic regulation and expression regulation [28]. The integration of transcriptomics and metabolomics offers a comprehensive characterization of cellular responses and helps to reveal the mechanisms of action of toxicants [29]. Utilizing these two approaches, it was found that polystyrene MPs caused endothelial cell (EC) injury and led to abnormal changes in alanine, aspartate, glutamate, and sphingolipid metabolism [30]. Similarly, multi-omics techniques revealed that human hepatic cells are affected by the toxicity of anthracene and its chlorides [31]. These studies demonstrate that multi-omics analyses are effective in identifying and linking molecules affected by chemical substances, revealing the underlying toxicological mechanisms.

In this study, we utilized a multi-omics approach to investigate the toxicity of PVC-MPs toward BEAS-2B cells, a respiratory cell line that is a major exposure target and toxicity model for MPs [19,32,33]. We revealed the key factors of PVC-MPs affecting cytotoxicity by integrating transcriptomics and metabolomics data.

## 2. Materials and Methods

### 2.1. PVC-MPs Characterization

PVC-MPs were purchased from Xingxiang New Materials Co., Ltd. (Dongguan, China). The morphology of PVC-MPs was examined via scanning electron microscopy (SEM) (SU5000, Hitachi, Japan). The average hydrodynamic size and zeta potential of PVC-MPs were measured using a Malvern Zetasizer Nano ZSP (Malvern Panalytical Ltd., Malvern, PA, USA).

### 2.2. Cell Culture and Cytotoxicity Testing

The BEAS-2B cell line was purchased from the American Type Culture Collection (ATCC). Cells were cultured at 37 °C and in 5% CO_2_ in a complete medium containing 10% fetal bovine serum (FBS), 4.5 g/L of D-glucose and L-glutamine, and 110 mg/L of sodium pyruvate. The effects of different concentrations of PVC-MPs on BEAS-2B cell viability after 24 h of exposure were assessed using a CCK-8 Cell Counting Kit (Vazyme, A311-02, Nanjing, China). The CCK-8 assay is more convenient and sensitive than the NRU assay and MTT assay. In this assay, the optical density (OD) value of methylated waste is measured at 450 nm using an enzyme marker, allowing for rapid assessment of cellular activity [34,35]. However, the CCK-8 assay can only be performed at a single time point, and colored drugs may interfere with the readings [36].

### 2.3. Transcriptomics Analysis

Total RNA was extracted from BEAS-2B cells in treated (800 μg/mL) and control groups with three biological replicates (*n* = 3) using TRIzol Reagent (LifeTechnologies, Carlsbad, CA, USA) according to the manufacturer’s instructions. The concentration and integrity of RNA were determined using a NanoDrop 2000 (Thermo Fisher Scientific, Wilmington, DE, USA) and an Agilent Bioanalyzer 2100 system (Agilent Technologies, Santa Clara, CA, USA) to detect the concentration, purity, and integrity of RNA. A total of 1 μg per sample was used to start library construction. Then, sequencing libraries were generated using the Hieff NGS Ultima Dual-mode mRNA Library Prep Kit for Illumina (Yeasen Biotechnology (Shanghai) Co., Ltd., Shanghai, China) with a dual-mode approach: firstly, mRNA enrichment with magnetic beads was used to enrich mRNA; then, USER enzyme was used to cut the hairpin loop structure; and, finally, PCR amplification and magnetic beads were used for purification. Paired-end sequencing was performed by using the Illumina NovaSeq platform to generate a 150-bp sequence. Differential expression analysis was performed on both groups using DESeq2, differentially expressed genes were identified using a negative binomial distribution model, and *p*-values were corrected using the Benjamini and Hochberg method. Differentially expressed genes (DEGs) were screened for fold change ≥1.5 and *p*-value < 0.05. Functional and pathway analyses of DEGs were conducted using the GO and KEGG databases.

### 2.4. Untargeted Metabolomics Analysis

Metabolites were extracted from BEAS-2B cells and divided into treated (800 μg/mL) and control groups with 6 biological replicates each (*n* = 6). To the samples, 1000 uL of extraction solution (methanol, acetonitrile, and water = 2:2:1 (*v*/*v*)) containing an isotope-labeled internal standard mixture was added. The samples were frozen in liquid nitrogen for 1 min and then thawed and vortexed at 4 °C for 30 s. The procedure was repeated 2–3 times, followed by sonication in an ice-water bath for 10 min, resting at −40 °C for 1 h, and centrifugation at 12,000 rpm for 15 min at 4 °C, and the supernatant was extracted for the assay. A Waters ACQUITY UPLC BEH Amide column was used as the chromatographic column on a Vanquish ultra-performance liquid chromatograph. The primary and secondary mass spectral data were obtained using an Orbitrap Exploris 120 mass spectrometer. Data were converted to the appropriate format using ProteoWizard software (Palo Alto, CA, USA), and peak localization, peak extraction, peak alignment, and integration were performed using the R program package. The data were normalized using internal standards (ISs). Data were logarithmically (LOG) transformed and centered (CTR) using SIMCA software (V16.0.2, Sartorius Stedim Data Analytics AB, Umea, Sweden). VIP > 1 and *p*-value < 0.05 were used as criteria to screen for differentially expressed metabolites (DEMs) between groups. Pathway enrichment analysis was performed using the MetaboAnalyst 5.0 platform (http://www.metaboanalyst.ca/) (accessed on 20 March 2024). 

### 2.5. Multi-Omics Analysis

Transcriptomics and metabolomics data were jointly analyzed using the Joint Pathway Analysis Module of MetaboAnalyst 5.0, and *p*-value < 0.05 was used as a screening criterion for significant enrichment of pathways. Metabolome–gene networks were displayed using Metascape software 3.5.

### 2.6. Statistical Analysis

The data were statistically analyzed using Zetasizer software (version 7.01) and GraphPad Prism software (version 10.2.3), and the results were presented as means ± SDs. Differences were assessed using the Student’s *t*-test or one-way analysis of variance (ANOVA) with Tukey’s post hoc test. *p*-value < 0.05 was considered significant.

## 3. Results

### 3.1. Characterization of PVC-MPs

**Figure 1:**
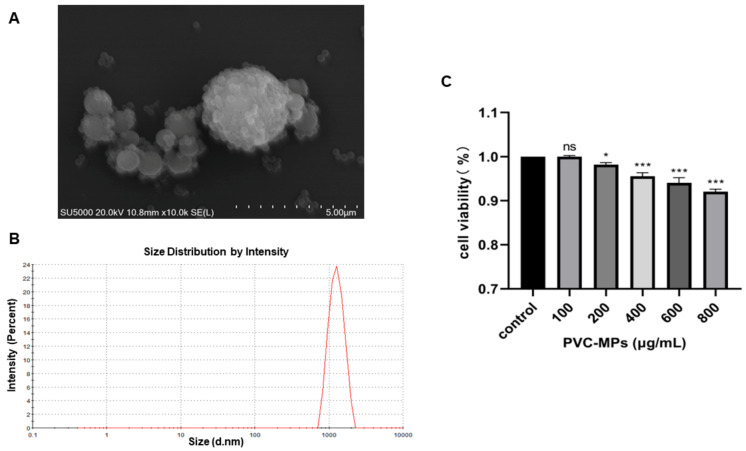
Characterization of PVC-MPs in suspension. (**A**) SEM images of PVC-MPs. (**B**) The physicochemical characterization of particle size. (**C**) Change in cell viability after exposure to different concentrations of PVC-MPs for 24 h. Results are shown as means ± SDs (*n* = 3 samples per treated group). ns (non-significant); * *p*-value < 0.05; *** *p*-value < 0.001.

In this study, we examined the morphology, size, and zeta potential of PVC-MPs to characterize them. The SEM images showed that PVC-MPs were spherical and aggregated into different sizes (Figure 1A). The average hydrodynamic size of the PVC-MPs in the medium was 1232 ± 70 nm (Figure 1B). The specific characterization results regarding the zeta potential of PVC-MPs are shown in Table 1. PVC-MPs of different concentrations showed negative charges in DMEM medium, indicating that they tend to repel each other and do not auto-aggregate.

### 3.2. Cytotoxicity Effects of PVC-MPs on BEAS-2B Cells

We evaluated the toxicity of PVC-MPs toward BEAS-2B cells with different doses (100, 200, 400, 600, and 800 μg/mL) for 24 h. As shown in Figure 1C, cell viability experiments showed that PVC-MPs significantly induced cytotoxicity at 200 μg/mL in a dose-dependent manner (*p*-value < 0.05). Overall, the above results indicated that the PVC-MPs adversely affected the BEAS-2B cells.

### 3.3. Transcriptomics Analysis of BEAS-2B Samples Exposed to PVC-MPs

#### 3.3.1. Screening and Analysis of Differentially Expressed Genes 

**Figure 2:**
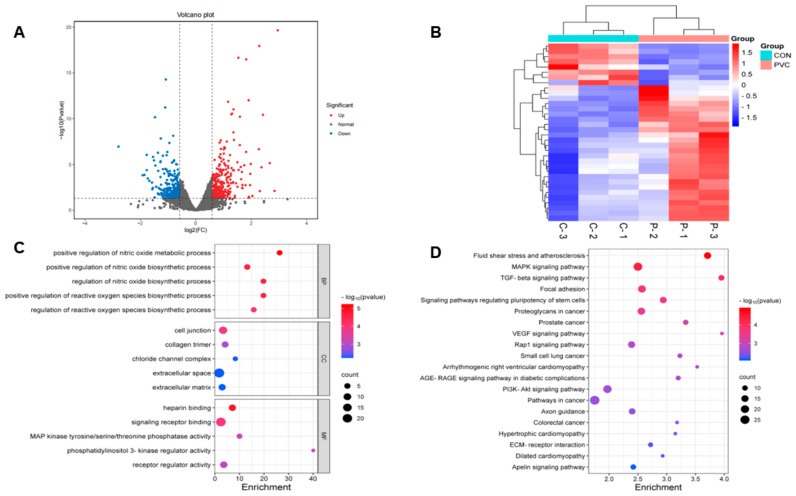
Transcriptomic analysis of BEAS-2B cells after their exposure to 800 μg/mL of PVC-MPs for 24 h. (**A**) Volcano plot of DEGs (blue, downregulated genes; red, upregulated genes). (**B**) Hierarchical clustering based on DEGs (blue, downregulated; red, upregulated). (**C**) GO enrichment analysis of DEGs. (**D**) KEGG pathway enrichment analysis of DEGs.

In this study, transcriptomic techniques were employed to analyze the gene expression changes in BEAS-2B cells following their exposure to PVC-MPs. After screening, we obtained a total of 530 DEGs, of which 282 were up-regulated and 248 were down-regulated (Figure 2A). The results showed that PVC-MPs had a significant effect on gene expression in BEAS-2B cells. Euclidean clustering analysis of the DEGs showed that there was a significant difference in gene expression patterns between the PVC-MP-exposed and control groups (Figure 2B).

#### 3.3.2. GO and KEGG Analysis

Gene Ontology (GO) analysis, a gene ontology-based method, categorizes genes into biological processes (BPs), cellular components (CCs), and molecular functions (MFs), aiding in the understanding of gene functions and interactions [37]. Figure 2C shows the enrichment of DEGs in the three GO categories. In the BP category, DEGs were mainly enriched in positive regulation of the nitric oxide metabolic process and positive regulation of the reactive oxygen species biosynthetic process. In the CC category, DEGs were mainly enriched in cell junction and collagen trimer, and in the MF category, DEGs were mainly enriched in heparin binding and signaling receptor binding. Furthermore, to explore the relationship between DEGs and cellular functions, we performed an enrichment analysis of the Kyoto Encyclopedia of Genes and Genomes (KEGG) pathway. KEGG is a database that collects and provides chemical, genomic, and functional information about biological systems, enabling the annotation of gene functions and metabolic pathways [38]. In this analysis, the q-value was used to indicate enrichment significance, with a lower q-value denoting higher significance. The results showed that DEGs were mainly involved in 20 pathways, among which the fluid shear stress and atherosclerosis pathway was the most highly enriched (Figure 2D). In addition, the MAPK signaling pathway and TGF-beta signaling pathway were also significantly enriched.

### 3.4. Metabolomics Analysis of BEAS-2B Samples Exposed to PVC-MPs

#### 3.4.1. Multivariate Analysis

**Figure 3:**
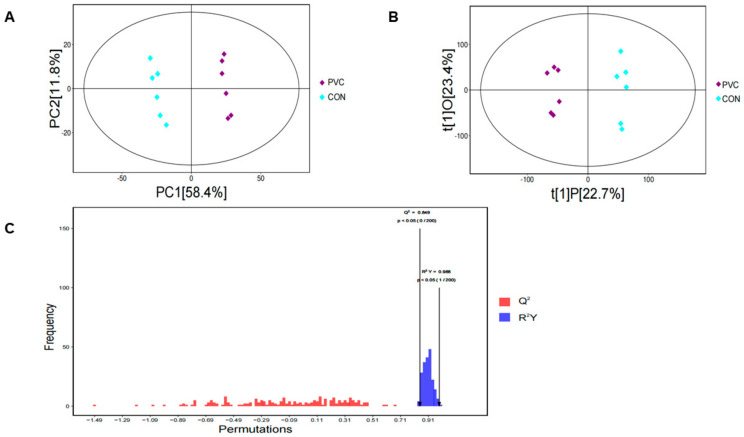
Multivariate analysis of metabolomics data on BEAS-2B cells after 24 h of exposure to PVC-MPs. (**A**) Scatter plot of PCA for metabolomics data. (**B**) Plot of OPLS-DA scores for metabolomics data. (**C**) Plot of the results of the permutation test for OPLS-DA modeling.

In this study, we analyzed the control and treated groups using untargeted metabolomics. First, we downscaled the data using principal component analysis (PCA) to show the overall characteristics of the data and sources of variation. As shown in Figure 3A, the PCA scatterplot clearly showed the differences between the two sample groups. Then, we used OPLS-DA to screen for metabolites associated with categorical variables. Similarly, the OPLS-DA plot showed significant differences between the PVC-MP metabolomics dataset and the control group (Figure 3B). Finally, we verified the quality of the model using a permutation test (*n* = 200). The results showed that the OPLS-DA model exhibited values of Q^2^ = 0.849 and R^2^Y = 0.988 (Figure 3C), indicating that the model had high stability and reliability.

#### 3.4.2. Screening and Analysis of Differentially Expressed Metabolites 

**Figure 4:**
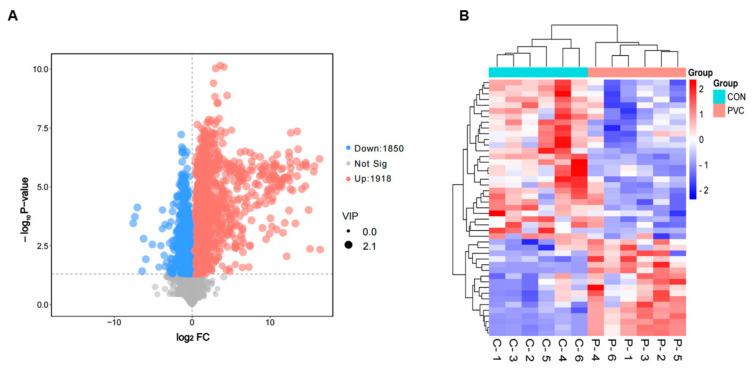
DEMs of BEAS-2B cells affected by exposure to PVC-MPs. (**A**) Volcano plot of DEMs (blue, downregulated metabolites; red, upregulated metabolites). (**B**) Hierarchical clustering of DEMs (blue, downregulated; red, upregulated).

We used a *p*-value < 0.05 and VIP > 1 as screening criteria for DEMs and used volcano plots to demonstrate metabolite changes and significance. As shown in Figure 4A, 3768 DEMs were significantly changed, among which 1918 were up-regulated and 1850 were down-regulated. We also analyzed the expression patterns of DEMs using the Euclidean distance matrix and fully interlocked clustering and found that there were significant differences between groups (Figure 4B).

#### 3.4.3. Metabolic Pathway Analysis

**Figure 5:**
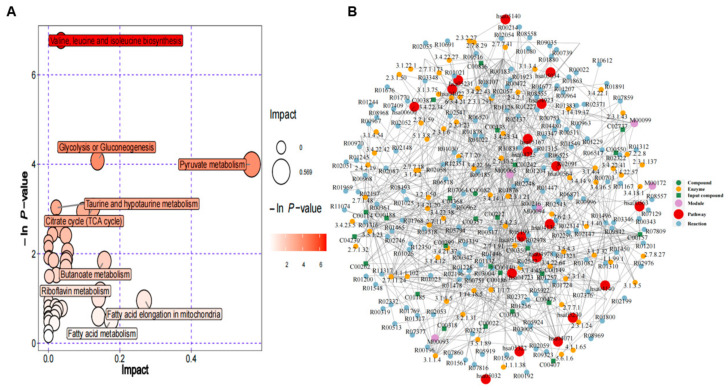
Pathway analysis of BEAS-2B cells exposed to PVC-MPs. (**A**) KEGG pathway enrichment analysis of DEMs. Bubble color indicates the *p*-value of enrichment analysis, and bubble size indicates the size of influencing factors in topology analysis. (**B**) Diagram of regulatory network analysis.

Using the KEGG Pathway database, we performed enrichment analysis of DEMs and used bubble plots to show the enrichment results regarding the metabolic pathways (Figure 5A). The results showed that these DEMs were enriched in 43 pathways (Table S1). Of these, valine, leucine, and isoleucine biosynthesis; glycolysis or gluconeogenesis; and pyruvate metabolism were the top three significantly enriched pathways, all of which are related to amino acid metabolism. To further explore the interactions between metabolic pathways, we also conducted a network enrichment analysis based on DEMs, including metabolic pathways, modules, enzymes, reactions, and metabolites (Figure 5B), reflecting the interactions and effects occurring between metabolic pathways as well as the propagation and targeting of perturbations at the pathway level.

### 3.5. Integrated Analysis of Transcriptomics and Metabolomics

**Figure 6:**
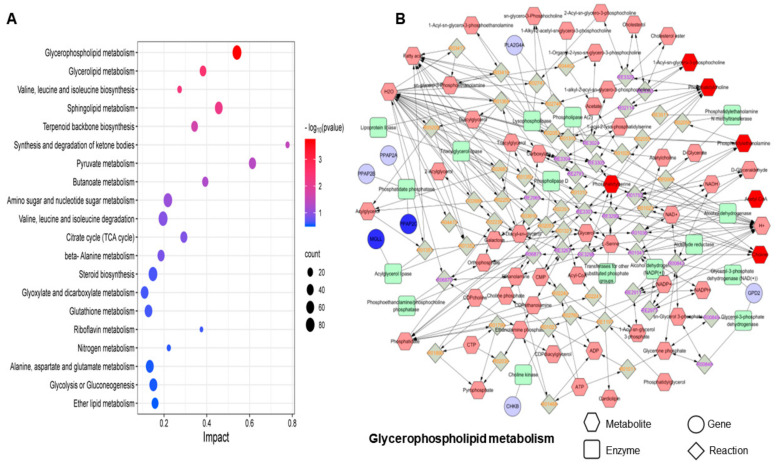
Association analysis of multi-omics data. (**A**) Joint pathway analysis of DEMs and DEGs using MetaboAnalyst 5.0. (**B**) Metabolite–gene network for glycerophospholipid metabolism (from Metscape).

**Figure 7:**
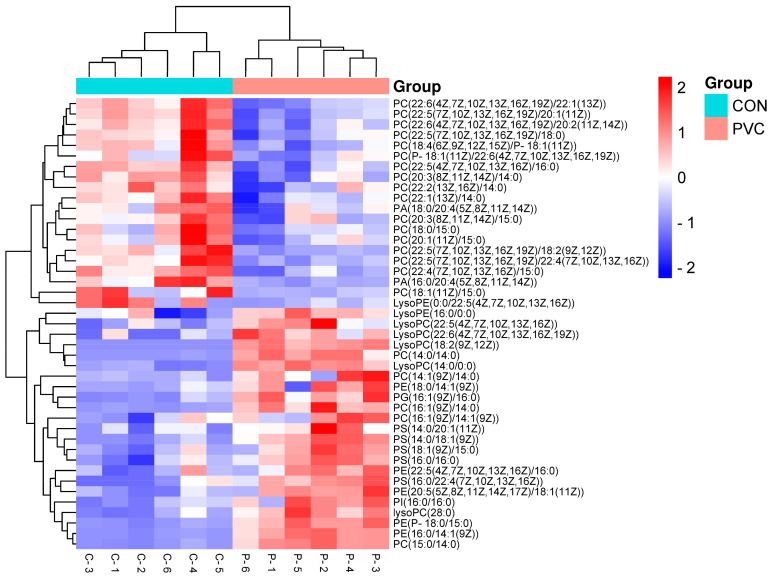
Heatmap of 43 target glycerophospholipids generated via Euclidean clustering analysis.

To explore the biological significance of DEGs and DEMs, we utilized the Joint Pathway Analysis module of MetaboAnalyst 5.0 for a comprehensive analysis. This analysis revealed key metabolic pathways in PVC-MP-exposed BEAS-2B cells. We calculated the *p*-value for each pathway using the hypergeometric test and illustrated the top 20 metabolic pathways that DEGs and DEMs jointly mapped to, as shown in Figure 6A. This analysis identified significant involvement of DEGs and DEMs in seven metabolic pathways (Table 2, *p* < 0.05): glycerophospholipid metabolism; glycerolipid metabolism; valine, leucine, and isoleucine biosynthesis; sphingolipid metabolism; terpenoid backbone biosynthesis; synthesis and degradation of ketone bodies; and pyruvate metabolism. Among these, glycerophospholipid metabolism was particularly perturbed, prompting us to construct and visualize the metabolome–gene network for this pathway using Metscape software (version 4.08) (Figure 6B). Additionally, we analyzed changes in matching metabolites within glycerophospholipid metabolism by generating a heat map through Euclidean clustering analysis (Figure 7).

## 4. Discussion

Microplastics, emerging environmental pollutants, are widespread worldwide. Studies have shown that airborne microplastic particles are capable of entering human lung tissue [39]. The total amount of microplastics ingested and inhaled by humans from the environment can be as high as 700–1050 μg per week [40]. In addition, numerous reports indicate that microplastics may contribute to the development of lung diseases, especially in individuals exposed to high levels over long periods of time [41]. Therefore, considering the total number of MPs accumulated and ingested in the human body over a long period of time, we chose 800 μg/mL of PVC-MPs as the exposure concentration for our experiment. At this concentration, BEAS-2B cells were exposed to PVC-MPs, and a detailed exploration of the specific effects of PVC-MPs at the cellular molecular level was conducted through high-throughput RNA sequencing and untargeted metabolomics analysis. We found that PVC-MPs could induce a decrease in cell viability in a dose-dependent manner. Previous studies have shown that PVC particles induce apoptosis in various cell types, such as normal human lung fibroblast cells (IMR 90) [42], enterocytes and hepatocytes [43], BHK-21 cells [44], and human lymphocytes [45]. Apoptosis has been reported to be a complex process regulated by multiple cell-signaling pathways, involving the expression and function of numerous genes and proteins [46]. Our study highlighted that the MAPK signaling pathway and the TGF-beta signaling pathway are the primary pathways through which PVC-MPs induce cellular responses. These two signaling pathways play pivotal roles in proliferation, differentiation, and apoptosis across various cell lines [47,48,49,50,51]. TGF-beta regulates the transcription of target genes by binding to their specific receptors and activating downstream SMAD proteins [52]. Meanwhile, there is clear crosstalk between the TGF-β and MAPK pathways and SMAD [53]. A study has demonstrated that both MAPK- and TGF-β-related signaling pathways are activated in pristine graphene-treated cells, leading to macrophage apoptosis [46]. In addition, in PVC-MP-treated cells, we observed an increase in the number of apoptotic genes in the MAPK signaling pathway and TGF-beta signaling pathway, which confirms that PVC-MPs may affect apoptosis in BEAS-2B cells at the transcriptome level.

Metabolomics analysis revealed the effects of environmental pollutants on organisms, in which endogenous metabolites, as end products of gene expression, directly reflect abnormal phenotypes of organisms [54]. In this study, we identified 3768 DEMs in PVC-MP-induced BEAS-2B cells, which were mainly involved in regulating amino acid metabolism. Amino acid metabolism can affect cellular metabolism and cellular processes at multiple levels, involving multiple metabolic pathways and regulatory mechanisms [55]. In particular, the branched-chain amino acid (BCAA) biosynthetic pathway plays an important role in protein synthesis and cell growth regulation [56,57]. Research has demonstrated that BCAA can promote the survival of eukaryotic cells and prolong the lifespan of Saccharomyces cerevisiae [58]. Furthermore, glycolysis and gluconeogenesis serve as the primary pathways for the cellular utilization and production of glucose, a crucial energy source. During glycolysis, glucose is metabolized into pyruvate, which can either enter the mitochondria to engage in the tricarboxylic acid (TCA) cycle, producing acetyl coenzyme A in the presence of oxygen, or be converted into lactate anaerobically through lactate dehydrogenase [59]. However, many diseased cells rely on aerobic glycolysis, known as the “Warburg effect” [60]. A study found that excessive glycolysis led to mitochondrial dysfunction and promoted the production of reactive oxygen species (ROS) [61], which led to cellular oxidative stress and consequently affected cellular autophagy and apoptosis [62]. Another report showed that elevated levels of leucine, isoleucine, valine, and phenylalanine in a Mycobacterium tuberculosis (MTB)-infected C57Bl/6 mouse model suggested that disorders of amino acid metabolism may be associated with alterations in multiple metabolic pathways [63]. These results suggest that disrupted amino acid metabolism may lead to imbalanced energy metabolism and apoptosis.

Multi-omics analysis is essential for understanding the biological mechanisms of diseases and identifying biomarkers by revealing the interactions between genes, proteins, metabolites, and microbiota [64]. This analytical approach dominates the study of cellular function and has enabled the systematic and comprehensive elucidation of complex biological processes by integrating different levels of biomolecular data [65]. In this study, the integrated transcriptomics and metabolomics analysis conducted revealed that lipid metabolism, encompassing glycerophospholipid metabolism, glyceride metabolism, and sphingolipid metabolism, was the most critical pathway for metabolic changes in BEAS-2B cells following exposure to PVC-MPs. Previous studies have demonstrated that lipid metabolism is closely linked to processes such as cell growth, apoptosis, and inflammation [66], influencing the characteristics of cell membranes, leading to the onset and progression of several diseases, including cancer [67]. Glycerophospholipid (GPL) is the major structural lipid of cell membranes [68], and its synthesis and metabolism in eukaryotes involve a variety of intermediates, such as phosphatidylcholine (PC), phosphatidylethanolamine (PE), and lysophosphatidic acid (LPA), which play important roles in cell signaling [69]. Moreover, alterations in GPL levels are important biological indicators of lipid metabolism disorders [70]. In this study, we found that exposure to PVC-MPs resulted in disturbed GPL metabolism in BEAS-2B cells, as evidenced by fluctuations in the content of multiple glycerophospholipids in PC and PE intermediates, as shown in Figure 7.

Environmental factors have had an important impact on lipid metabolism, with air pollution, as an important environmental factor, being capable of disturbing lipid metabolism, leading to lipid peroxidation, oxidative stress, and inflammatory responses, which can increase the risk of developing chronic diseases [71]. Disturbances in GPL metabolism have been observed following gastrointestinal exposure to airborne PM2.5 [72], as well as perturbations in arachidonic acid and glycerolipid metabolism due to the exposure of human bronchial epithelial cells to PM [73]. Furthermore, ceramide, a key molecule in sphingolipid metabolism, has been shown in various IR models to be strongly associated with apoptosis induced by mitochondrial damage [74]. Recent studies have also demonstrated that abnormal sphingolipid metabolism induces apoptosis in a variety of cells, including CNE-2 cells and breast cancer cells [75,76,77]. In this study, we found that the levels of sphingomyelin (SM) were upregulated in sphingolipid metabolic pathways, including SM (d16:1/24:1(15Z)), SM (d18:1/12:0), SM (d18:0/14:0), and SM (d18:1/14:0). SM is a key sphingolipid essential for processes such as apoptosis, proliferation, and migration and plays a central role in maintaining plasma membrane stability and signaling [78,79]. Additionally, PC, PE, SM, and cholesterol constitute the main components of biological membranes [80]. In this study, exposure to PVC-MPs resulted in changes in PC, PE, and SM levels in BEAS-2B cells, indicating possible damage to the cell membrane that could affect cell survival and metabolic processes.

There are several limitations of our present study. Primarily, MPs are encountered as intricate mixtures in the environment [81]. Our methodology involved the utilization of a singular concentration and type of MPs, which might not encapsulate the comprehensive spectrum of biological responses elicited by varying concentrations and types of MPs on BEAS-2B cells. Additionally, the cytotoxicity evaluation executed via the CCK-8 assay potentially neglected the detection of MPs at diminished concentrations. Thirdly, while pivotal biological pathways were delineated through multi-omics analysis, an in-depth exploration of the specific mechanisms governing these pathways was not conducted. Collectively, these limitations indicate the direction of our future research.

## 5. Conclusions

We analyzed changes in the transcriptome and metabolome of BEAS-2B cells after their exposure to PVC-MPs. Through a comprehensive analysis of transcriptomics and metabolomics data, we identified disruptions of lipid metabolism in PVC-MP-exposed BEAS-2B cells. The results reveal that PVC-MPs interfere with the metabolic mechanism of BEAS-2B cells and provide new potential targets for the prevention and treatment of PVC-MP-induced lung diseases.

## Figures and Tables

**Table 1:** Zeta potentials of different concentrations of PVC-MP dispersions in DMEM medium.

Concentration (μg/mL)	Zeta Potential (mV)
100	−27.53
200	−25.17
400	−25.10
600	−25.52
800	−31.83

**Table 2:** Significant enrichment pathways for DEGs and DEMs.

KEGG ID	Pathway	*p*-Value
ko00564	Glycerophospholipid metabolism	0.0001756
hsa00561	Glycerolipid metabolism	0.0033649
ko00290	Valine, leucine, and isoleucine biosynthesis	0.0046556
map00600	Sphingolipid metabolism	0.0068775
map00900	Terpenoid backbone biosynthesis	0.021066
ko00072	Synthesis and degradation of ketone bodies	0.034016
ko00620	Pyruvate metabolism	0.043594

## Data Availability

The data presented in this study are available on request from the corresponding author.

## Supplementary Materials

The following supporting information can be downloaded at https://www.mdpi.com/article/10.3390/toxics12060399/s1. Table S1: DEMs enrichment pathways.
